# Myxoma Virus dsRNA Binding Protein M029 Inhibits the Type I IFN-Induced Antiviral State in a Highly Species-Specific Fashion

**DOI:** 10.3390/v9020027

**Published:** 2017-02-02

**Authors:** Masmudur M. Rahman, Grant McFadden

**Affiliations:** The Biodesign Institute, Center for Immunotherapy, Vaccines, and Virotherapy, Arizona State University, Tempe, AZ 85287-5401, USA; Masmudur.Rahman@asu.edu

**Keywords:** Myxoma virus, M029, Poxvirus, type I IFNs, antiviral state, dsRNA binding protein, PKR

## Abstract

Myxoma virus (MYXV) is a *Leporipoxvirus* that possesses a specific rabbit-restricted host tropism but exhibits a much broader cellular host range in cultured cells. MYXV is able to efficiently block all aspects of the type I interferon (IFN)-induced antiviral state in rabbit cells, partially in human cells and very poorly in mouse cells. The mechanism(s) of this species-specific inhibition of type I IFN-induced antiviral state is not well understood. Here we demonstrate that MYXV encoded protein M029, a truncated relative of the vaccinia virus (VACV) E3 double-stranded RNA (dsRNA) binding protein that inhibits protein kinase R (PKR), can also antagonize the type I IFN-induced antiviral state in a highly species-specific manner. In cells pre-treated with type I IFN prior to infection, MYXV exploits M029 to overcome the induced antiviral state completely in rabbit cells, partially in human cells, but not at all in mouse cells. However, in cells pre-infected with MYXV, IFN-induced signaling is fully inhibited even in the absence of M029 in cells from all three species, suggesting that other MYXV protein(s) apart from M029 block IFN signaling in a species-independent manner. We also show that the antiviral state induced in rabbit, human or mouse cells by type I IFN can inhibit M029-knockout MYXV even when PKR is genetically knocked-out, suggesting that M029 targets other host proteins for this antiviral state inhibition. Thus, the MYXV dsRNA binding protein M029 not only antagonizes PKR from multiple species but also blocks the type I IFN antiviral state independently of PKR in a highly species-specific fashion.

## 1. Introduction

Evasion of host innate immune responses is one of the key steps for successful replication and pathogenesis for all viruses that infect vertebrate hosts. Type I interferons (IFNs) play a central role in inducing early antiviral responses to combat most virus infections [1,2]. The synthesis of type I IFN ligands is initiated upon innate sensing of viruses and their virus-induced pathogen-associated molecular patterns (PAMPs) by cellular pattern recognition receptors (PRRs). Activation of PRRs induces signaling cascades that lead to the rapid expression of genes encoding antiviral cytokines, such as the type I IFNs. Induced IFNs are then secreted and bind their cognate receptors on either the same virus-infected cells (autocrine stimulation) or on neighboring uninfected cells (paracrine stimulation) to initiate the IFN signaling cascade that leads to the formation of a transcriptional activation complex called ISGF3, which finally upregulates a large suite of IFN-stimulated genes (ISGs). The summated activities of many dozens or even hundreds of induced ISGs then operationally mediate the antiviral state that limits the progression of the virus infection. However, the capacity of the IFN-induced antiviral state to block any given virus varies greatly depending on not only the virus anti-IFN strategies, but also the cell type and species of the infected host.

Large DNA viruses like poxviruses inhibit these IFN-dependent antiviral responses at multiple levels. Like many poxviruses, Myxoma virus (MYXV), a member of the *Leporipoxvirus* genus, exhibits a very restricted host range in nature within the lagomorph family and is totally nonpathogenic in hosts outside rabbit species [3,4]. In European rabbits (*O. cuniculis*), MYXV causes a unique lethal disease called myxomatosis, in which essentially all of the host innate and acquired immune pathways are rendered ineffectual. However, in contrast, MYXV exhibits a much broader cellular tropism for cultured transformed cell lines derived from many diverse mammalian species outside the rabbit [5]. Antiviral cytokines, such as tumor necrosis factor (TNF) or Type I IFN, are known to play significant roles in the restriction of MYXV replication in primary cells from non-rabbit hosts [6]. For example, primary mouse fibroblasts are nonpermissive for MYXV replication because of the rapid induction of type I IFN in response to MYXV infection, which then mediates an antiviral state that the virus cannot overcome [7]. The critical role of type I IFN in protection against MYXV infection in mice was further confirmed by the observation that mice lacking signal transducer and activator of transcription 1 (STAT1) are susceptible to lethal MYXV infection after intracranial inoculation [7]. On the other hand, primary human fibroblasts are permissive to MYXV replication and do not induce IFN as a consequence of the infection, but these cells can be induced to become nonpermissive after pre-treatment with type I IFN and/or TNF [6,8]. Interestingly, primary human macrophages are nonpermissive for MYXV because these cells do induce type I IFN as a consequence of retinoic acid-inducible gene I (RIG-I) sensing of the MYXV infection [9].

Since MYXV is currently being developed as oncolytic virus for many classes of human cancers, a more detailed understanding of how MYXV responds to IFN activities in different host species and in cell types of varying lineages will help to provide more mechanistic explanations for why the virus is utterly rabbit-specific in nature, but also possesses an apparently expanded tropism for cancer cells and tissues in mice and humans [10,11,12].

Poxviruses encode dozens of proteins that have evolved to counteract the various host innate antiviral immune responses, such as those induced by the type I IFNs [13,14]. For example, multiple vaccinia virus (VACV) proteins have been identified that antagonize IFN responses at many levels, such as minimizing the sensing of viral PAMPs, neutralizing the IFN ligands by preventing their binding to IFN receptors, blocking the Janus kinase/signal transducer and activator of transcription (JAK/STAT) signal transduction pathway after IFN receptor activation, and finally the more global inhibition of elements of the antiviral state induced by multiple ISGs [15,16]. For example, three of the known vaccinia virus host range genes, namely *E3L*, *K1L* and *C7L* also antagonize antiviral activities induced by type I IFNs. VACV lacking expression of *E3L* or both *K1L* and *C7L* are defective for replication in many mammalian cell lines [17,18]. Mechanistically, *K1L* and *C7L* antagonize the antiviral activities induced by IFN-regulated factor (IRF) 1 [19]. Interestingly, *C7L* orthologs from many other poxviruses are able to rescue deletions of VACV *C7L*. In some cases, a single viral protein may possess anti-IFN activities at multiple levels. For example, the VACV double-stranded RNA (dsRNA) binding protein E3 blocks the activation of both IRF3 and IRF7 [20], nuclear factor κB (NF-κB) [21,22] and ISG15 [23], all of which contribute to the antiviral state after IFN treatment. Thus, deletion of the *E3* gene of VACV results in increased sensitivity to the inhibitory effects of IFNs [24]. However, the in vitro and in vivo roles of E3 and related dsRNA binding proteins from other pathogens differ significantly. For example, replacing E3 with dsRNA binding proteins from unrelated viruses or bacteria or even from other poxviruses can only restore some host range functions of E3 in cell culture but not in vivo pathogenesis [25]. This suggests that dsRNA-binding or E3-like proteins have acquired unique host specific immune modulatory functions, depending on their evolutionary origins.

We recently reported that MYXV-encoded dsRNA binding protein, M029—a truncated relative of VACV E3-like proteins—plays a key role for permissive MYXV replication in essentially all mammalian cells tested in culture, as well as functions as a critical virulence factor for myxomatosis in European rabbits [26]. M029 lacks the significant portion of the N-terminal “Z-DNA binding domain” of the VACV E3 protein, which is required by VACV for the inhibition of the type I IFN response in mice and in mouse embryo fibroblasts (MEFs) [27]. At the molecular level, M029 binds and inhibits cellular protein kinase R (PKR) in a dsRNA-dependent manner in order to subvert PKR-dependent antiviral responses, even in mammalian cells derived from multiple non-rabbit species, including humans and mice [26]. In addition to PKR as a host cell target, M029 also interacts with another innate cellular protein called RHA/DHX9, a member of the DEXD/H box family of helicases, in a dsRNA-independent manner to promote MYXV replication in human cells [26]. Since dsRNA binding proteins have been shown to have role in the inhibition of host innate responses against viruses, we proposed that M029 will play a key role against cellular IFN responses. The inhibition of type I IFN responses by VACV E3 is mediated by the blockade of PKR activation [21]. However, unlike VACV, the cellular tropism for MYXV in human or mouse cells can be dramatically affected by the extent of induced type I IFN responses [6,8]. A recent study demonstrated that replacing VACV E3 with MYXV M029 rescues only some of the in vitro functions of E3 but not the in vivo pathogenesis, which suggests that these two related viral proteins might in fact possess distinct cellular targets for the modulation of antiviral innate immune responses [25].

In this study, we now demonstrate that M029 plays a major role in the species-specific inhibition of type I IFN-induced antiviral state against MYXV. MYXV successfully antagonizes essentially all of the rabbit IFN pathways in rabbit cells, and M029 is critical for counteracting the IFN-induced antiviral state. However, M029 only partially antagonizes the human type I IFN antiviral state in human cells and is totally unable to antagonize the murine type I IFN antiviral state in mouse cells. In contrast, in pre-infected cells from all three species, MYXV is able to effectively inhibit type I IFN-induced signaling downstream of IFN receptor activation in a fashion that is independent of M029. Importantly, MYXV sensitivity to type I IFN-induced antiviral state in human and mouse cells cannot be rescued even in the absence of PKR. These results demonstrate that the MYXV dsRNA binding protein M029 blocks IFN-induced antiviral pathways and also mediates species-specific permissiveness in cells, but via both PKR-dependent and –independent pathways. M029 effectively inhibits PKR in cells from multiple species, and thus contributes to the permissive replication of MYXV in many classes of human cancer cells. In addition, M029 also antagonizes the type I IFN-induced antiviral state independently of PKR, but in a highly rabbit-specific fashion, this at least partially accounts for the inability of this virus to infect primary cells and tissues from non-rabbit hosts.

## 2. Materials and Methods

### 2.1. Cell Lines and Cell Culture

Rabbit cell line RK13 (ATCC# CCL-37), RK13 expressing VACV E3 protein (RK13-E3; [26], human cell lines HeLa (ATCC# CCL-2), HeLashControl and HeLashPKR [26], primary human fibroblasts GM02504 (Coriell Institute for Medical Research, Camden, NJ, USA), mouse embryonic fibroblast cell lines NIH3T3 (ATCC# CRL-1658), monkey cell line BSC-40 (ATCC# CRL-2761) all were cultured in Dulbecco minimum essential medium (DMEM; GIBCO, Thermo Fisher Scientific, Waltham, MA, USA) supplemented with 10% fetal bovine serum (Atlanta Biologicals, Flowery Branch, Georgia, USA), 2 mM glutamine (Invitrogen, Thermo Fisher Scientific) and 100 µg of penicillin-streptomycin/mL (GIBCO). WT-MEF and MEF PKR-/- cells (donated by Dr. Robert Silverman of Lerner Research Institute, Cleveland, Ohio, USA) were cultured in RPMI 1640 medium (BioWhittaker, Lonza, Basel, Switzerland) supplemented with 10% FBS, and 100 µg of pen/strep per mL. All cultures were maintained at 37 °C in a humidified 5% incubator. Transfection of PKR siRNA (Santa Cruz Biotechnology, Dallas, Texas, USA) in RK13 cell line using Lipofectamine RNAiMAX (Invitrogen) was performed as described before [26].

### 2.2. Construction of Recombinant Viruses and Viral Preparation

Construction of wild-type (WT)-MYXVs expressing different reporter proteins vMyx-GFP (WT-MYXV expressing GFP under a poxvirus synthetic early/late promoter), vMyx-Fluc (WT-MYXV expressing the firefly luciferase protein (Fluc) under a poxvirus synthetic early/late promoter) and vMyx-GFP-TdTomato (WT-MYXV expressing GFP under a poxvirus synthetic early/late promoter and TdTomato under a poxvirus p11 late promoter) were described before [6,28,29]. Construction of vMyx-M029KO virus was described previously [26,30]. Vesicular stomatitis virus (VSV) expressing GFP was prepared as described before [31]. A recombination plasmid having reporter genes for Fluc driven by a synthetic early/late (sE/L) poxvirus promoter and tandem-dimer tomato red fluorescent protein (Tr-FP) driven by poxvirus p11 late promoter flanked by M135 and M136 gene locus [29] of MYXV was used to make the vMyx-M029KO-Fluc virus. Briefly, RK13-E3 cells were infected with the vMyx-M029KO virus for one hour and then the cells were transfected with the recombination plasmids. Multiple rounds of foci purifications were performed on the same cell lines based on TdTomato-Red and GFP expression and continued until pure foci were isolated. Recombination was confirmed by polymerase chain reaction (PCR) using appropriate primers (data not shown). The M029-minus viruses were grown and amplified in RK13-E3 cells. All other myxoma viruses were grown in BSC40 or RK13 cells.

### 2.3. Interferon (IFN) Sensitivity and Virus Replication Assays

Cells were seeded in multiwell dishes the day before infection with 60%–70% confluency. The following day cells were treated individually with hIFNβ/mIFNβ/uIFNα (PBL Assay Science, Piscataway, NJ, USA; 500 U/mL) or rabbit IFN containing media [26] for 18 h and then infected with the viruses with different multiplicity of infection (MOI) depending on the experiments. One hour post-infection (hpi), the media were removed, washed and incubated with fresh medium containing no or same concentration of IFN that was added. For post-IFN treatment, the cells were first infected with the viruses for one or six hours (h), the media were removed, washed and incubated with the IFN containing media. For microscopy, at 24 or 48 hpi, the infected cells were visualized under an inverted fluorescence microscope and photographed with a digital camera. For viral replication assays, cells were harvested at various times post-infection and stored in −80 °C until processed. Before titration cells were freeze-thawed at −80 °C and 37 °C for three times and sonicated for one minute to release the viruses from infected cells. Depending on the viruses, they were tittered back either RK13 or RK13-E3 cells by serial dilution.

### 2.4. Luciferase Assay

Fluc activity was measured to monitor viral gene expression in different cell lines under different treatment conditions. The assay was performed using 96-well plate. The cells were seeded with 80%–100% confluency and allowed to adhere for overnight or 6–8 h after seeding. For pretreatment with IFN the complete media was replaced with media containing IFNs (500 U/mL) and incubated for 18 h. For virus infections, viruses were added directly in the media in the presence or absence of IFN based on the MOI to be used and allowed the infection for different time periods. Luciferase assay was performed using the luciferase reporter assay kit (Promega, Madison, WI, USA), following manufacturer instructions. Briefly, media was removed at the indicated time points, cells were washed with phosphate buffered saline (PBS) once and added the lysis buffer. Lysis was done at RT for 15–20 mins and substrate was added and reading was taken immediately after adding the substrate using a microplate reader, Appliskan (Thermo Fisher Scientific).

### 2.5. RNA Purification and Real-Time Polymerase Chain Reaction (qPCR)

For isolation of RNA, 1 × 10^6^ cells were plated in each well of six-well dishes. The following day cells were mock treated, treated with IFNs or infected with the viruses at a MOI of 5. For post-IFN, cells were first infected with the viruses for six hours and the media was replaced with IFN containing fresh media. In all cases cells were harvested 24 h after infection or treatment with IFNs. Total RNA isolation, cDNA preparation and real-time PCR (qPCR) were performed based on the protocol described before [6]. The PCR reactions were run on an ABI 7300 qPCR machine under the following conditions: 95 °C for 10 min, followed by 40 cycles of 95 °C for 15 s and 60 °C for 1 min. Primers used for qPCR analysis are: human GAPDH (F: GTGGACCTGACCTGCCGTCT, R: GGAGGAGTGGGTGTCGCTGT), human ISG15 (F: CGCAGATCACCCAGAAGATCG, R: TTCGTCGCATTTGTCCACCA) human Mx1 (F: CAGCACCTGATGGCCTATCA, R: ACGTCTGGAGCATGAAGAACTG), mouse GAPDH (F: AGGTCGGTGTGAACGGATTTG, R: TGTAGACCATGTAGTTGAGGTCA), mouse ISG15 (F: GGTGTCCGTGACTAACTCCAT, R: TGGAAAGGGTAAGACCGTCCT), mouse Mx1 (F: GACTACCACTGAGATGACCCAGC, R: ATTTCCTCCCCAAATGTTTTCA), rabbit GAPDH (F: GTGGACCTGACCTGCCGCCT, R: AGAGGAGTGGGTGGCACTGT), rabbit Mx1 (F: CTCATCAGCCTGGAGGTCAG, R: CCTGATGAGCGCCTTGATCT), rabbit OAS1 (F: TCCGGTTCCTCTGCATCTAC, R: GCCTTGAGCTGTTTCCTGAC). Amplification of genes was normalized to GAPDH amplification from the same sample and the fold induction of genes after viral infection or IFN treatment was calculated relative to the unstimulated control of the cell line.

### 2.6. Western Blot Analysis

Western blot analysis was performed as described before [26]. Briefly, the mock or virus infected cells were collected at different time points after infection, washed with PBS and processed with RIPA lysis buffer. Equal amounts of total proteins were used for Western blot analysis. The membranes were first probed with primary antibody, washed, incubated with secondary antibody, again washed and detected using the chemiluminescence substrate and exposure to X-ray film. The detection antibodies were as follows: rabbit antibodies against phospho-STAT1 and rabbit polyclonal antibodies against STAT1 (Cell Signaling Technology, Danvers, MA, USA); mouse monoclonal antibodies against actin (Ambion, Thermo Fisher Scientific); mouse monoclonal antibodies against PKR (Santa Cruz Biotechnology); rabbit antibodies against ISG15 (Cell Signaling Technology). Generation of rabbit polyclonal and mouse monoclonal antibodies against MYXV proteins M-T7 and Serp-1 was described before [32,33].

### 2.7. Generation of Knockout Cells Using CRISPR/Cas9

HeLa cells with an inactivated PKR gene were generated by using the CRISPR/Cas9 plasmid (Santa Cruz Biotechnology) according to the manufacturer’s instructions. After transfection, cells were treated with puromycin (2 μg/mL) for 48 h. The surviving cells were plated in 96-well plates with ~1–5 cells/well. Colonies were expanded and were selected according to their ability to support replication of vMyxM029KO virus.

### 2.8. Statistical Analysis

Data were expressed as means ± SD and were analyzed by paired *t*-test. Significant difference was accepted at *p* < 0.05.

## 3. Results

### 3.1. Species-Specific Inhibition of Type I IFN-Induced Antiviral States by Myxoma Virus (MYXV)

**Figure 1:**
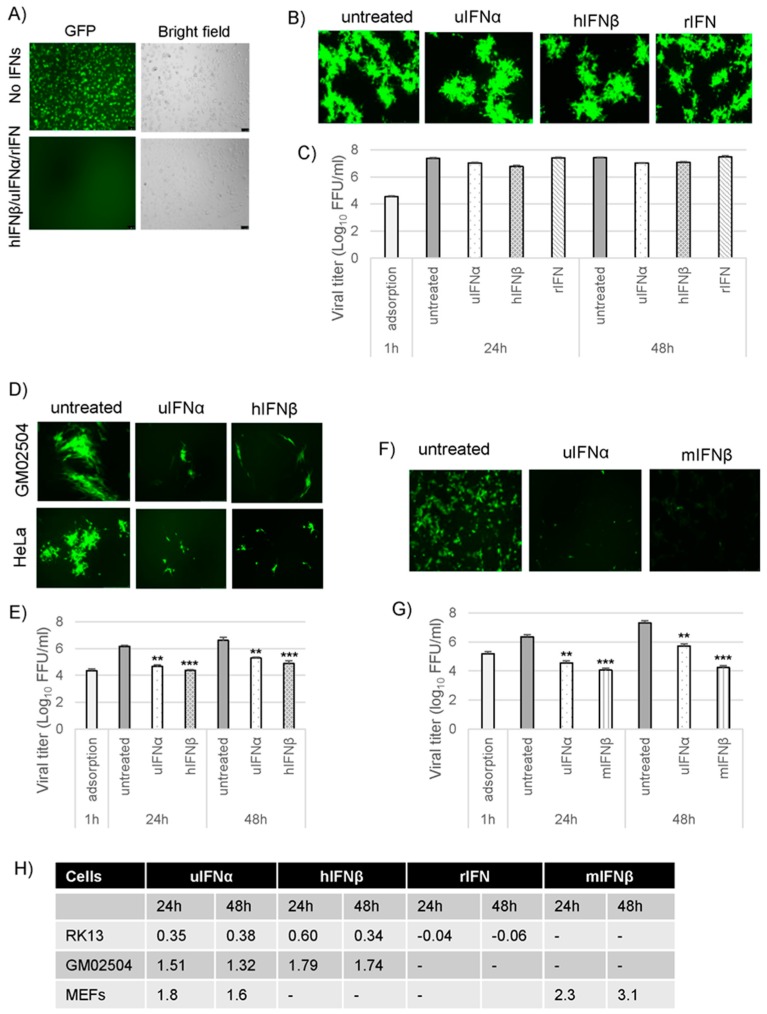
Myxoma virus (MYXV) is able to inhibit the type I interferon (IFN)-induced antiviral state completely in rabbit cells, partially in human cells and poorly in mouse cells. RK13 cells were individually treated with uIFNα, hIFNβ or rIFN containing media for 18 h and (**A**) infected with vesicular stomatitis virus (VSV)-GFP at a multiplicity of infection (MOI) of 1.0, fluorescence images were taken 48 h post-infection (hpi) and a representative image is shown; scale bars (100 µm); (**B**) infected with the wild-type (WT)-MYXV at an MOI of 0.01 and fluorescence images were taken 48 hpi; (**C**) infected with the wild-type (WT)-MYXV at an MOI of 5.0 and then cells were collected at 1 h (adsorption), 24 h and 48 hpi for virus titration; (**D**) Human GM02504 primary fibroblasts or HeLa cells were treated with uIFNα or hIFNβ containing media for 18 h and infected with the WT-MYXV at an MOI of 0.01 and fluorescence images were taken 48 h after infection; (**E**) GM02504 cells were mock or treated with uIFNα or hIFNβ containing media for 18 h and infected with the WT-MYXV at an MOI of 5.0 and then cells were collected at 1 h (adsorption), 24 h and 48 h after infection for virus titration; (**F**) Mouse embryo fibroblast (MEF) cells were treated with uIFNα or mIFNβ containing media for 18 h and infected with the WT-MYXV at an MOI of 1.0 and fluorescence images were taken 48 h after infection; (**G**) MEF cells were mock or treated with uIFNα or mIFNβ containing media for 18 h and infected with the WT-MYXV at an MOI of 5.0 and then cells were collected at 1 h (adsorption), 24 h and 48 hpi for virus titration. The virus titers were determined in triplicate following serial dilution onto RK13 cells. Statistics are relative to untreated virus titer after 24 h or 48 h infection. ** *p* < 0.01 *** *p* < 0.001; (**H**) Table showing log_10_ changes in virus titer after different type I IFN treatment compare to the untreated samples at 24 h and 48 h in RK13, GM02504 and MEFs.

The type I IFN-induced antiviral states in cells originated from different species can have differential effects on the productive replication of MYXV. In order to examine whether MYXV is able to overcome the antiviral states in the Type I IFN treated cells, representative species specific cell lines RK13, GM02504/HeLa and MEF derived from rabbit, human and mouse, respectively were tested. RK13 cells were pretreated with universal IFNα (uIFNα), human IFNβ (hIFNβ) or rabbit IFN (rIFN) for 18 h and then infected with the GFP-tagged wild type (WT) MYXV, vMyx-GFP, at an MOI of 0.01 for monitoring virus spread via foci formation or an MOI of 5 for checking single-cycle progeny virus formation. In RK13 cells, treatment with any of these type I IFNs completely blocked the replication of control VSV virus, suggesting that antiviral states induced by these type I IFNs are functional (Figure 1A). However, unlike VSV, the WT-MYXV was able to replicate and form foci normally in the presence of any of these type I IFNs (Figure 1B). When checked for progeny virus formation at 24 and 48 hpi, the WT-MYXV in the type I IFN treated cells, was also able to produce similar levels of progeny just like infection without any IFN treatment (Figure 1C). This was further confirmed by calculating the log_10_ changes in virus titers (Figure 1H, top row), where any changes of less than 0.6 log is regarded as marginal. These results indicate that in rabbit RK13 cells, MYXV is able to inhibit all aspects of the type I IFN-induced antiviral state to allow permissive virus replication.

We then checked whether in human and mouse cell lines, the type I IFN treatment-induced antiviral states can be overcome by MYXV like in rabbit RK13 cells. Human primary fibroblasts GM02504 or transformed HeLa cell lines were pretreated with uIFNα or hIFNβ for 18 h and infected with WT-MYXV at an MOI of 0.01 for foci formation or MOI of 5 for progeny virus formation. Pre-treatment with either uIFNα or hIFNβ retarded, but did not completely inhibit, the virus spread and foci formation in both GM02504 and HeLa cells (Figure 1D). As expected, the formation of progeny virus was also significantly reduced but not completely inhibited under these treatment conditions in the tested human cell lines, suggesting that MYXV is partially able to inhibit the type I IFN treatment-induced antiviral state in human cells (Figure 1E). When we calculated log_10_ changes in virus titer in response to IFN pre-treatment of human GM02504 cells, hIFNβ reduced virus titer slightly more than uIFNα but both can be considered to be effective inhibitors of MYXV (Figure 1H, middle row). Similar to rabbit and human cell lines, immortalized MEFs were pretreated with uIFNα or mIFNβ for 18 h and infected with WT-MYXV for monitoring infection and progeny virus formation. In MEFs, although both the type I IFNs reduced MYXV gene expression and replication, pretreatment with mIFNβ completely inhibited progeny virus formation, unlike the human or rabbit cells (Figure 1F,G). This was further confirmed by comparing the log_10_ changes in virus titer after type I IFN treatments (Figure 1H, lower row).

### 3.2. M029 is Required for the Global Inhibition of Type I IFN-Induced Antiviral States in Rabbit Cells

**Figure 2:**
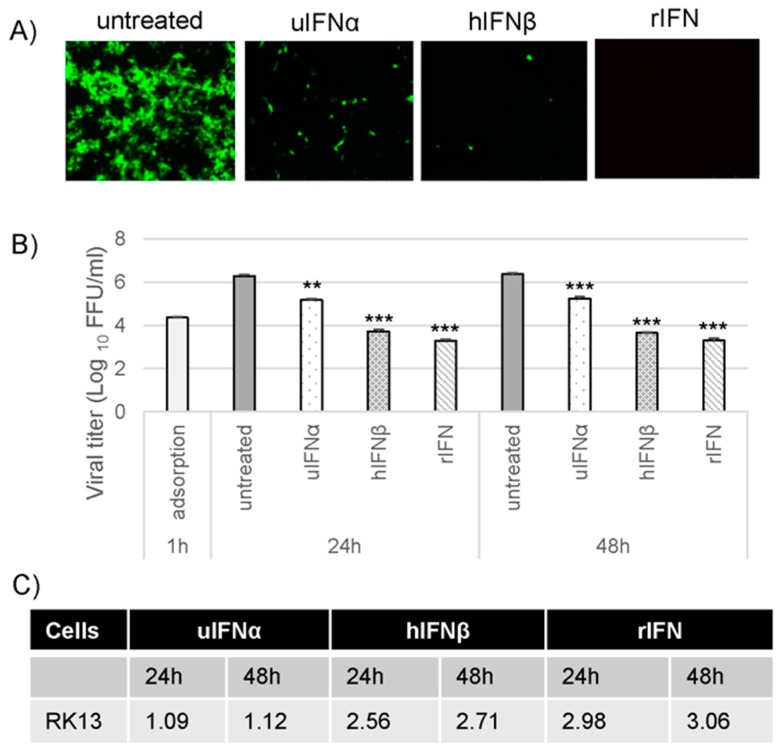
M029 is required for the complete inhibition of the type I IFN-induced antiviral state in rabbit RK13 cells. (**A**) RK13 cells were treated with uIFNα, hIFNβ or rIFN containing media for 18 h and infected with the vMyxM029KO virus at an MOI of 0.1 and fluorescence images were taken 48 hpi; (**B**) RK13 cells were mock or treated with uIFNα, hIFNβ or rIFN containing media for 18 h and infected with the vMyxM029KO virus at an MOI of 5.0 and then cells were collected at 1 h (adsorption), 24 h and 48 hpi for virus titration. The virus titers were determined in triplicate following serial dilution onto permissive RK13-E3 cells. Statistics are relative to untreated virus titer after 24 h or 48 hpi. ** *p* < 0.01 *** *p* < 0.001; (**C**) Table showing log_10_ changes in virus titer after different type I IFN treatment compare to the untreated samples at 24 h and 48 h in RK13.

Virus-encoded dsRNA-binding proteins, for example VACV E3, are known to antagonize multiple aspects of IFN-induced activities in infected cells [15]. Based on our observation that MYXV can inhibit type I IFN-induced antiviral states, but only in a highly species-specific manner, we anticipated that M029 might have a key role in the inhibition of IFN activities in rabbit cells. To test whether M029 is critical for the inhibition of IFN activities in rabbit cells, RK13 cells were pretreated with uIFNα, hIFNβ and rIFN for 18 h, and infected with the M029-minus virus, vMyxM029KO, in the continued presence of type I IFNs. Viral gene expression and foci formation was monitored using fluorescence microscopy after 48 hpi. Results indicated that vMyxM029KO virus was unable to replicate and form foci in the presence of any of the type I IFNs in RK13 cells (Figure 2A). This suggests that in rabbit cells M029 is required by MYXV to antagonize the type I IFN-induced antiviral state and for the subsequent establishment and spread of virus infection in neighboring cells. We also checked progeny virus formation at 24 h and 48 hpi with an MOI of 5. The results indicate that in the absence of M029, MYXV is unable to produce any significant levels of infectious progeny in the presence of hIFNβ or rIFN (Figure 2B). Under these treatment conditions, uIFNα also reduced the viral titer significantly in RK13 cells, albeit not as efficiently as the other IFNs, which was further evident after calculating the log_10_ changes in viral titer (Figure 2C). These results further indicate that M029 is required for the inhibition of the type I IFN-induced antiviral state in rabbit cells.

### 3.3. Loss of Protein Kinase R (PKR) Can Rescue M029-Minus MYXV Replication in Mouse Embryo Fibroblasts (MEFs) but Is Unable to Rescue Virus Resistance to the Mouse Type I IFN-Induced Antiviral State

**Figure 3:**
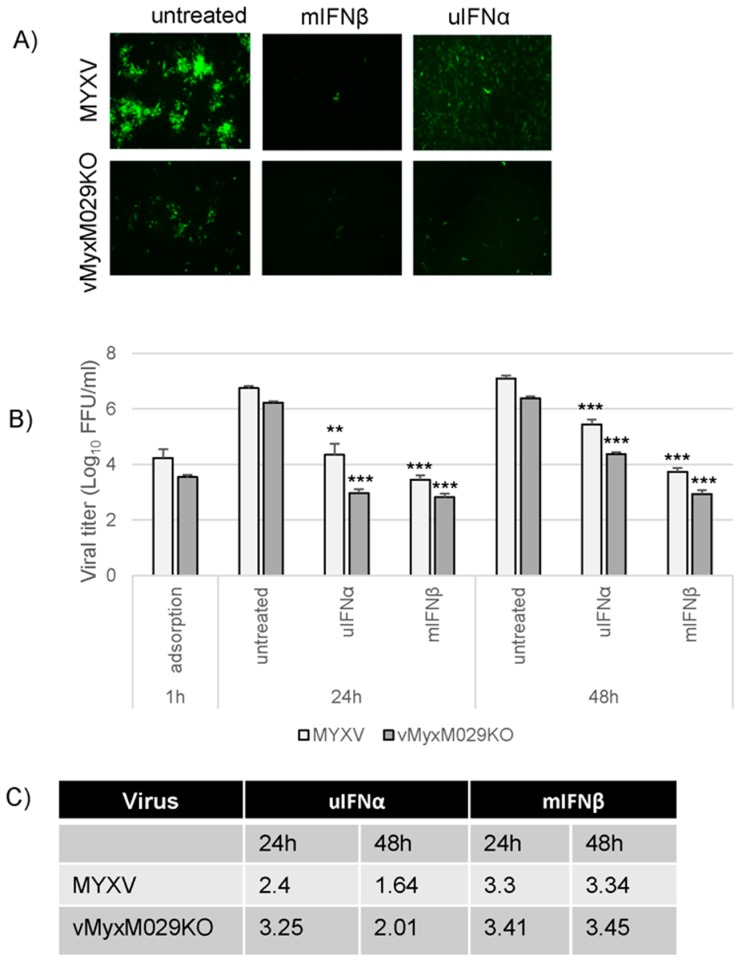
Loss of protein kinase R (PKR) can rescue M029-minus MYXV replication in mouse cells but not overcome sensitivity to the mouse type I IFN-induced antiviral state. (**A**) MEF PKR-/- cells were treated with uIFNα, or mIFNβ containing media for 18 h and infected with the WT-MYXV or vMyxM029KO virus at an MOI of 0.1 and fluorescence images were taken 48 hpi; (**B**) MEF PKR-/- cells were mock or treated with uIFNα or mIFNβ containing media for 18 h and infected with the WT-MYXV or vMyxM029KO viruses at an MOI of 5.0 and then cells were collected at 1 h (adsorption), 24 h and 48 hpi for virus titration. The virus titers were determined in triplicate following serial dilution onto permissive RK13-E3 cells. Statistics are relative to virus titer after 24 h or 48 h infection. ** *p* < 0.01 *** *p* < 0.001; (**C**) Table showing log_10_ changes in virus titer after different type I IFN treatment compare to the untreated samples at 24 h and 48 h in MEF PKR-/- cells.

Since M029-minus MYXV, vMyxM029KO, was unable to replicate in WT-MEFs or NIH3T3 murine cell lines [26], we tested vMyxM029KO virus replication in an engineered PKR-minus MEF cell line (MEF PKR-/-). The absence of PKR now partially rescued infection and replication of vMyxM029KO virus in these MEF PKR-/- cells, suggesting that M029 is competent for the inhibition of PKR even in mouse cells (Figure 3A). When MEF PKR-/- cells were pre-treated with uIFNα or mIFNβ, both WT-MYXV and M029-minus virus gene expression and infections were blocked to comparable degrees (Figure 3B). In the MEF PKR-/- cells both WT-MYXV and vMyxM029KO viruses were unable to produce any progeny when the cells were treated with mIFNβ prior to virus infection, although uIFNα had less inhibitory effects on progeny virus formation from both the viruses, as observed with other cell lines. This was also supported when we calculated the log_10_ changes in virus titer for both the viruses after treatment with either uIFNα or mIFNβ (Figure 3C). These results confirm that M029 is not capable of antagonizing the mouse type I IFN-induced antiviral state in mouse cells but is still required for the inhibition of murine PKR to allow viral replication and cellular tropism when IFN is absent.

### 3.4. M029 is Required for the Partial Inhibition of Type I IFN-Induced Antiviral States in Human Cells in Either the Presence or Absence of PKR

**Figure 4:**
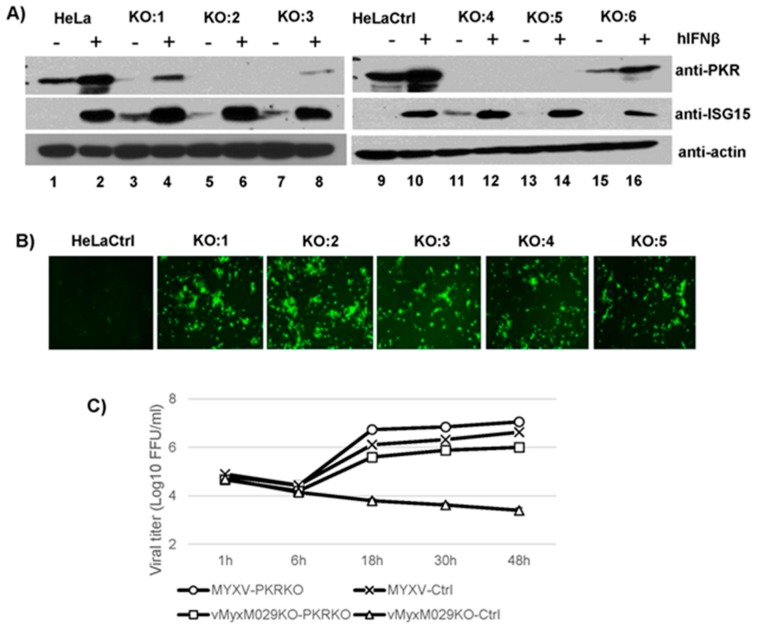
CRISPR-mediated PKR knockout in HeLa cells can rescue vMyxM029KO virus replication. (**A**) Generation of PKR knockout cells by CRISPR/CAS9 technology. HeLa cells were transfected with the CRISPR/Cas9 plasmids as described in Materials and Methods. Cells from different colonies (represented as KO) expanded, treated with hIFNβ for 18 h, lysed, and total proteins were resolved by SDS-PAGE and analyzed by Western blotting to detect endogenous PKR, IFN-stimulated gene 15 (ISG15) and actin as a loading control. Colony 2 (KO:2) was selected for further experiments and labeled as HeLa PKR-/- cells; (**B**) vMyxM029KO virus is able to replicate in the HeLa PKR-/- colonies. HeLa ctrl or HeLa PKR-/- colonies were infected with vMyxM029KO virus and fluorescence images were taken 48 hpi; (**C**) Single step growth curves of MYXV infection in HeLa ctrl and HeLa PKR-/- cells. The indicated cells were infected with WT-MYXV or vMyxM029KO at an MOI of 5, and then cells were collected at 1, 6, 18, 30 and 48 hpi. The virus titers were determined in triplicate following serial dilution onto RK13-E3 cells. Data are representative of three independent experiments.

MYXV can only partially inhibit human type I IFN activities in IFN-sensitive human cells. We next checked whether M029 played any role in this partial inhibition of type I IFN-induced antiviral state and whether PKR is involved in mediating this IFN-mediated antiviral activity against MYXV. We previously showed that M029-minus MYXV was able to replicate in human cells only when PKR level was reduced [26]. Since type I IFN treatment itself can stimulate PKR expression, even with shRNA-mediated PKR knockdown (data not shown), we have constructed PKR-knockout human HeLa cell lines using CRISPR technology, which respond to exogenous type I IFN, like the human fibroblasts cell line GM02504. We selected HeLa PKR-KO clones that did not express PKR before or after IFN treatment, however, expression of ISG15 was confirmed in these cells, indicating that type I IFN responses are still functional (Figure 4A). In these HeLa clones, the partially rescued replication of vMyxM029KO virus was also confirmed (Figure 4B). Analysis of virus replication using single step growth curves in HeLa PKR-/- and HeLa control cell lines, demonstrated that the titer of vMyxM029KO virus was significantly enhanced after PKR knockout (Figure 4C).

**Figure 5:**
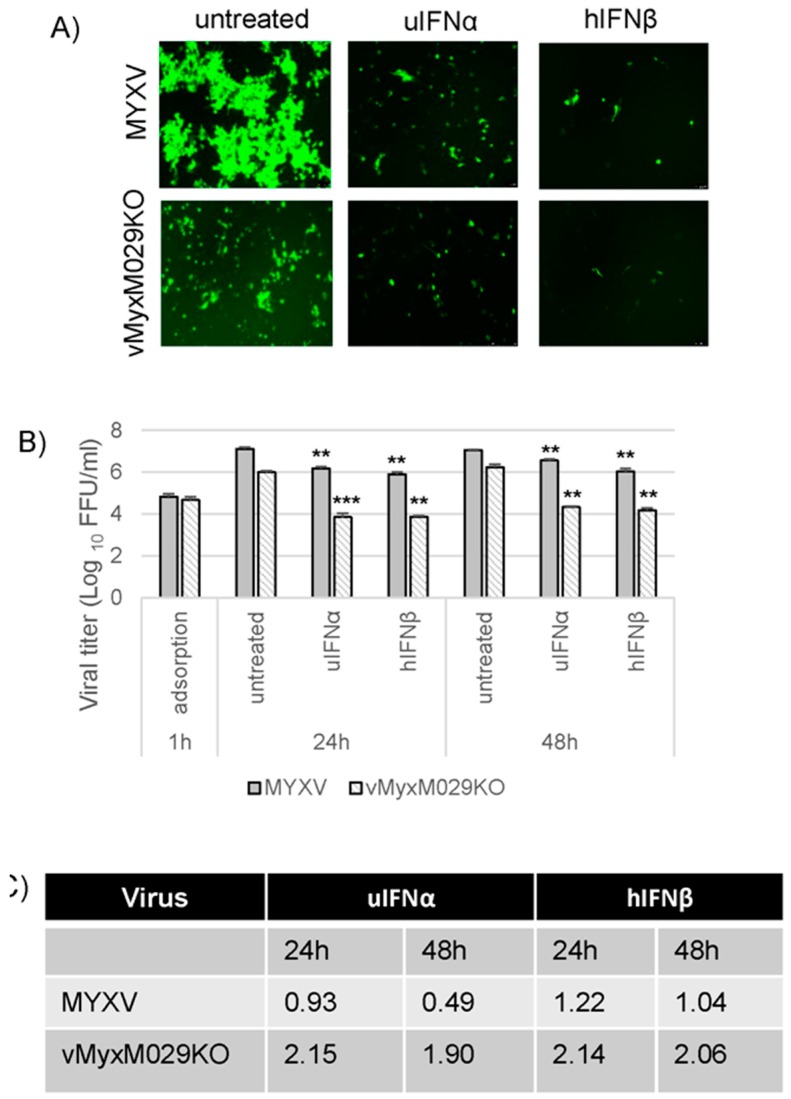
M029 is required for partial inhibition of the type I IFN-induced antiviral state in human cells; (**A**) HeLa PKR-/- cells were treated with uIFNα or hIFNβ containing media for 18 h and infected with the WT-MYXV or vMyxM029KO viruses at an MOI of 0.01 and fluorescence images were taken 48 hpi; (**B**) HeLa PKR-/- cells were mock or treated with uIFNα, hIFNβ containing media for 18 h and infected with the WT-MYXV or vMyxM029KO viruses at an MOI of 5.0 and then cells were collected at 1 h (adsorption), 24 h and 48 hpi for virus titration. The virus titers were determined in triplicate following serial dilution onto permissive RK13-E3 cells. Statistics are relative to untreated virus titer after 24 h or 48 h infection. ** *p* < 0.01 *** *p* < 0.001; (**C**) Table showing log_10_ changes in virus titer after different type I IFN treatment compare to the untreated samples at 24 h and 48 h in HeLa PKR-/- cells.

In addition, PKR knockout also enhanced WT-MYXV progeny titers. When HeLa PKR-/- cells were pretreated with uIFNα or hIFNβ and then infected with WT-MYXV or vMyxM029KO viruses at an MOI of 0.1 we observed that virus spread and foci formation was retarded (Figure 5A) suggesting that IFN-induced antiviral activity is partially functional against MYXV in human cells even in the absence of PKR. We also measured the formation of progeny virus in these uIFNα or hIFNβ-treated and MYXV or M029-minus virus-infected HeLa PKR-/- cells. As expected, both uIFNα and hIFNβ-induced antiviral responses reduced the titer of WT-MYXV in HeLa PKR-/- cells like parental Hela or GM02504 cell lines. However, under these IFN-treatment conditions, vMyxM029KO virus was unable to produce any progeny virus (Figure 5B). Again, hIFNβ had more potent antiviral effects based on the log_10_ changes in viral titer (Figure 5C). These results confirm that, in human cells, M029 is required to partially overcome the type I IFN induced antiviral state, but in a manner that is independent of PKR.

### 3.5. PKR Knockdown in Rabbit Cells Cannot Rescue vMyxM029KO Virus Replication after Establishment of the Type I IFN-Induced Antiviral State

**Figure 6:**
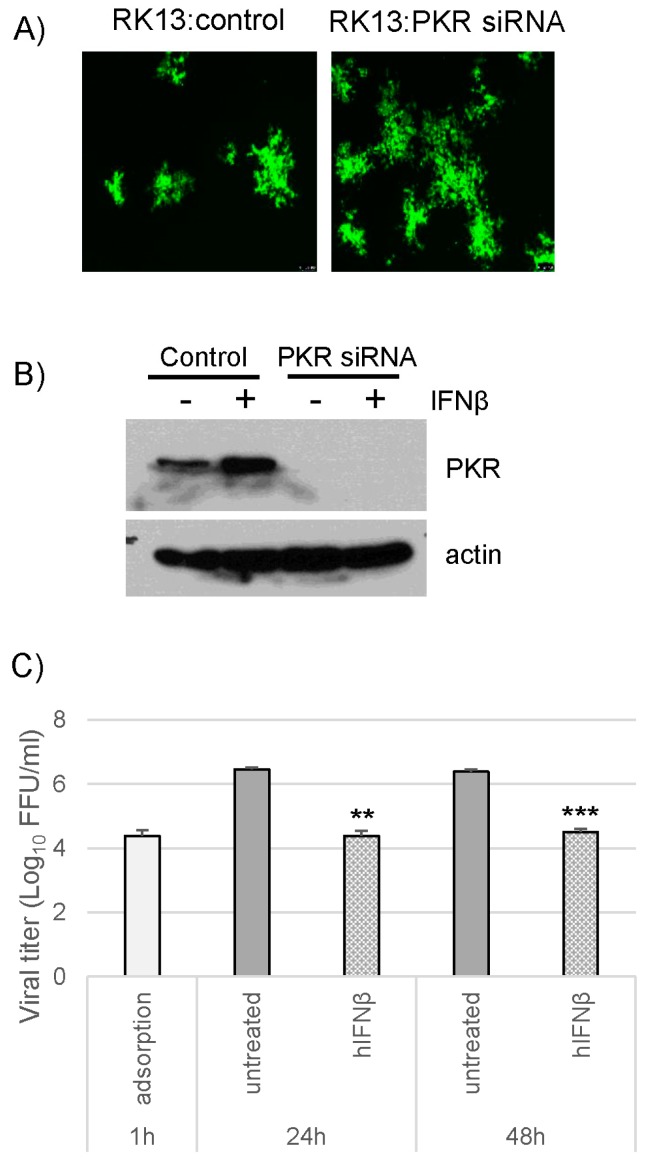
Knockdown of PKR cannot rescue M029-minus MYXV sensitivity to the type I IFN-induced antiviral state in rabbit RK13 cells. RK13 cells were transiently transfected with siRNAs targeting rabbit PKR for 48 h before infection with the viruses or treatment with hIFNβ; (**A**) Control or PKR knockdown RK13 cells were infected with vMyxM029KO virus at an MOI of 0.01 and fluorescence images were taken 48 hpi; (**B**) Western blot analysis of PKR knockdown RK13 cells to detect endogenous PKR and actin as loading control; (**C**) PKR knockdown RK13 cells were infected with vMyxM029KO viruses before or after treatment with hIFNβ at an MOI of 5 and then cells were collected 1 h, 24 h and 48 h for titration onto RK13-E3 cells. Statistics are relative to untreated virus titer after 24 h or 48 h infection. ** *p* < 0.01 *** *p* < 0.001.

Like human and mouse cells, we then tested the role of PKR in the type I IFN-induced antiviral states in rabbit RK13 cells by siRNA-mediated transient knockdown of PKR. We have confirmed that, at least in RK13 cells, type I IFN treatment after PKR knockdown did not further enhance the level of PKR protein as assessed using Western blot analysis (Figure 6B). Unlike human and mouse cells, PKR knockdown in RK13 cells did not enhance the titer of vMyxM029KO virus significantly as observed by foci formation and virus titration (Figure 6A and data not shown). When treated with type I IFN, the PKR knockdown RK13 cells were not able to support vMyxM029KO virus replication, like the parental RK13 cells (Figure 6C). These results again confirm that, like human and mouse cells, PKR in rabbit cells is not an essential effector target for the inhibition of type I IFN-induced antiviral state by MYXV.

### 3.6. Type I IFN-Induced Antiviral States Inhibit Viral Protein Synthesis in a Highly Species-Specific Manner

**Figure 7:**
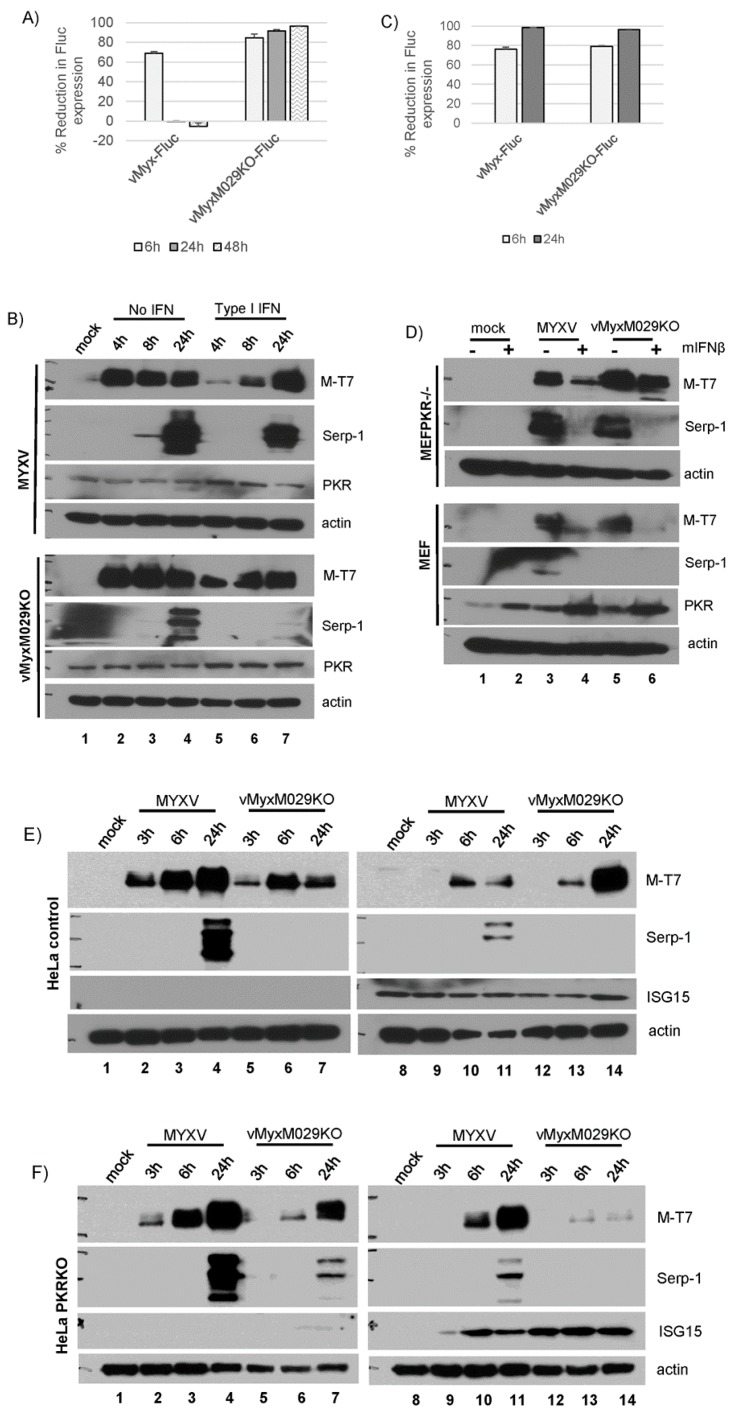
Type I IFN-induced antiviral state can inhibit the expression of MYXV early and late proteins in a species-specific manner; (**A**) Rabbit RK13 and (**C**) Mouse MEF PKR-/- cells were treated with hIFNβ and mIFNβ containing media for 18 h and infected with vMyx-FLuc (WT-MYXV) or vMyxM029KO-FLuc viruses for the indicated time points. Cells were then processed for luciferase assay. Percent reduction in FLuc expression was calculated from the samples not pretreated with IFNs. The assay was performed in triplicate; (**B**) Rabbit RK13 cells were treated with mock or hIFNβ containing media for 18 h and infected with WT-MYXV or vMyxM029KO viruses and cells were collected at the indicated time points for Western blot analysis; (**D**) Mouse MEFs and MEF PKR-/- cells were treated with mock or mIFNβ containing media for 18 h and infected with WT-MYXV or vMyxM029KO viruses and cells were collected at he indicated time points for Western blot analysis; (**E**) Human HeLa control and (**F**) HeLa PKR-/- cells were treated with mock (lanes 1-7) or hIFNβ (lanes 8-14) containing media for 18 h and infected with WT-MYXV or vMyxM029KO viruses and cells were collected at the indicated time points for Western blot analysis.

Our results indicate that the type I IFN-induced antiviral state can compromise MYXV replication, either in the absence or presence of M029, at multiple levels in various cell types originated from different species outside the rabbit. To monitor at what level the replication of WT-MYXV and M029-minus viruses are affected by IFN pre-treatment in cells from different species, we measured the level of viral gene expression using recombinant viruses expressing FLuc under poxvirus synthetic early/late promoter and monitored the expression of early and late proteins by western blot analysis. In all of these experiments, we have only used the species-specific type I IFNs that showed the most potent inhibition against MYXV replication. In rabbit RK13 cells, IFN pretreatment and infection with WT-MYXV caused an initial retardation in the initiation of viral gene expression at 6 h, but which was totally recovered by 24–48 h (Figure 7A). On the other hand, vMyxM029KO virus was unable to rescue viral gene expression even at the later time points of infection in the IFN-treated RK13 cells (Figure 7A). This suggests that de novo expression of M029 is required for the effective inhibition of the IFN-induced antiviral state in rabbit cells. When we analyzed early (M-T7) and late (Serp-1) viral protein synthesis by Western blot analysis in RK13 cells, type I IFN treatment had minimal effect on late protein synthesis of WT-MYXV, but viral late gene expression was completely lost when infected with vMyxM029KO virus (Figure 7B).

In contrast to RK13 cells, in WT-MEFs (data not shown) or PKR-/- MEF cells, pretreatment with mIFNβ almost completely blocked viral late gene expression at 6 h and 24 h, which was even more evident when infected with vMyxM029KO virus (Figure 7C). This was further confirmed by analyzing early and late protein synthesis by Western blot (Figure 7D). This observation clearly demonstrates that in MEFs, type I IFN-induced antiviral states block MYXV late protein synthesis even in the absence of PKR. We also monitored the effect of type I IFN-induced antiviral states on viral gene expression in HeLa control and HeLa PKR-/- cells using Western blot analysis. In the parental HeLa or HeLa control cells, replication of vMyxM029KO virus was blocked due to the lack of late gene expression (Figure 7E). In this cell line, type I IFN treatment significantly reduced both early and late protein synthesis of WT-MYXV. In the HeLa PKR-/- cell lines, late viral gene expression was rescued for vMyxM029KO virus infection, but which was completely inhibited by the treatment with type I IFN (Figure 7F). In contrast, the late gene expression of WT-MYXV was further reduced by type I IFN even in the absence of PKR. Collectively, these results indicate that M029 is unable to antagonize the mIFNβ-induced antiviral state in mouse cells, only partially in human cells, but does so very efficiently in rabbit cells.

### 3.7. MYXV Does Not Alter the Levels of Pre-Existing Type I IFN-Induced IFN-Stimulated Genes (ISGs) in Rabbit, Human or Mouse Cells

**Figure 8:**
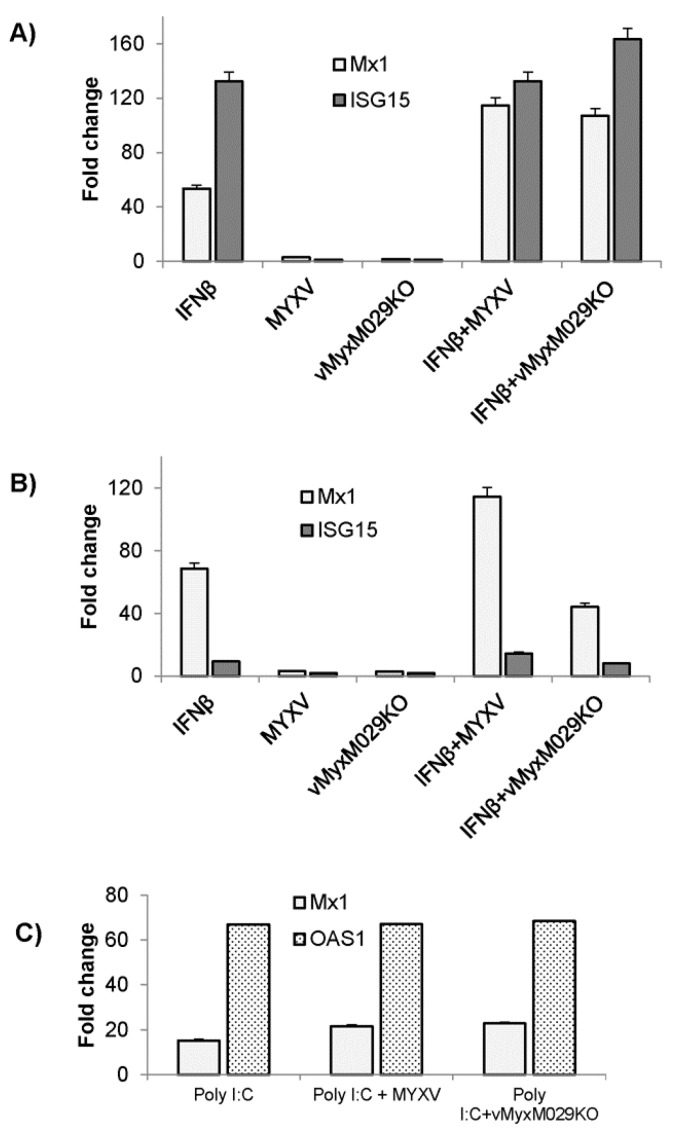
MYXV infection does not alter levels of ISGs in IFN pre-treated cells originating from different species; (**A**) Human HeLa PKR-/- and (**B**) Mouse MEF PKR-/- cells were treated with hIFNβ and mIFNβ respectively for 18h and infected with either WT-MYXV or vMyxM029KO viruses for another 24 h. Total RNA was isolated from the harvested cells and subjected to reverse transcription and real-time polymerase chain reaction (qPCR) to monitor the expression of ISGs. Fold changes are based on GAPDH as control; (**C**) Rabbit RK13 cells were transfected with poly I:C for 18 h and infected with either WT-MYXV or vMyxM029KO viruses for 24 h and total RNA was isolated from the harvested cells and subjected to reverse transcription and qPCR to monitor the expression of ISGs. Fold changes are based on GAPDH as control.

The next step was to check whether the failure of M029-minus virus to replicate in IFN-treated rabbit cells, and in PKR-deficient permissive human or mouse cells, can be explained by virus-induced changes in the levels of ISG proteins. The induction of antiviral ISGs, for example Mx1, ISG15, and OAS1 in response to IFN treatment was assessed in rabbit, human and mouse cells. Significantly increased ISG expression levels after IFN treatment were detected in human (HeLa) and mouse (MEF) cells, however, the upregulation of ISGs were relatively less robust in rabbit (RK13) cells in response to human or rabbit IFN treatment alone (Figure 8A,B, and not shown). However, transfection of poly I:C significantly enhanced the levels of induced expression of these ISGs in RK13 cells, which did not affect the WT-MYXV replication under these conditions (Figure 8C, and not shown). To test whether these levels of expressed ISGs were altered after WT-MYXV or vMyxM029KO virus infection, the cells were pre-treated with IFN or transfected with poly I:C (RK13 cells) and then infected with the test viruses. Measuring the levels of ISG RNAs by qPCR indicate that both the WT-MYXV and vMyxM029KO viruses were unable to alter the tested ISG mRNA levels in HeLa, MEF or RK13 (or by MYXV and M029KO virus alone) cell lines when the IFN is added prior to the virus. We also observed that infection with either virus alone did not enhance the transcription of any tested ISGs. These results suggest that neither WT-MYXV nor M029-minus virus alters the levels of pre-existing ISG that mediate the antiviral state in IFN pre-treated cells of any of the three species that were tested. We have also transiently expressed M029 alone in human cells and observed no inhibition in the levels of IFN-induced expression of tested ISGs (data not shown). In addition, we have not observed any effects on the IFN-induced expression of selective ISGs at the translational level in HeLa cells (data not shown). Collectively, these data indicate that the inability of M029-minus MYXV to replicate in human or mouse cells is not due to any inability to alter pre-expressed ISGs that mediate the antiviral state.

### 3.8. MYXV Can Inhibit Type I IFN Signaling Even in the Absence of M029 in Multiple Cell Species

**Figure 9:**
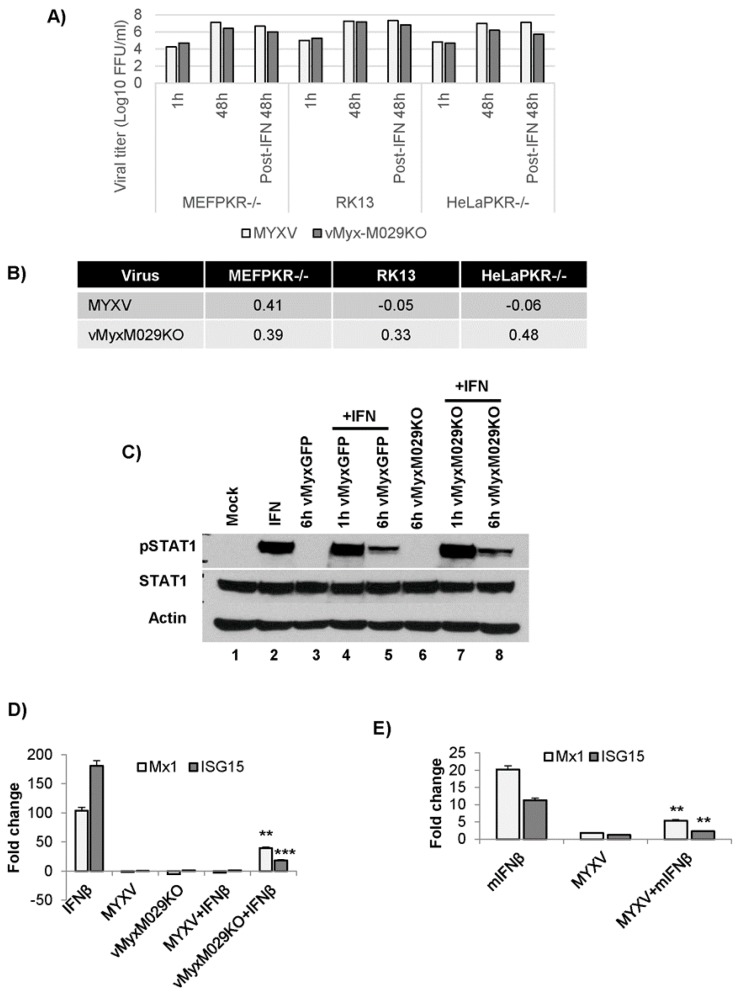
MYXV infection is able to inhibit post-infection type I IFN signaling even in the absence of M029; (**A**) MEF PKR-/-, RK13 and HeLa PKR-/- cells were infected with WT-MYXV and vMyxM029KO viruses alone for 1 h (adsorption) and 6 h, after which media containing mIFNβ or hIFNβ (500 U/mL) were added in one of the sample and harvested after 48 h for virus titration; (**B**) Table showing log_10_ changes in virus titer after different type I IFN treatment compare to the untreated samples at 48 h in MEF PKR-/-, RK13 and HeLa PKR-/- cells; (**C**) HeLa cells were left untreated, treated with hIFNβ for 30 mn, infected with vMyxGFP and vMyxM029KO viruses alone for 1 h and 6 h or treated with hIFNβ for 30 mn post-infection. The membranes were first probed with anti-pSTAT1 antibody, stripped and probed for STAT1 and again stripped and probed for actin (loading control); (**D**) HeLa PKR-/- cells were treated with hIFNβ for 18h after mock or infection with vMyxGFP or vMyxM029KO viruses for 6 h and total RNA was isolated from the cells and subjected to reverse transcription and qPCR to monitor the expression of Mx1 and ISG15. Fold changes are based on GAPDH as control; (**E**) MEF PKR-/- cells were treated with mIFNβ for 18 h after mock or infection with vMyxGFP for 6 h and total RNA was isolated from the cells and subjected to reverse transcription and qPCR to monitor the expression of Mx1 and ISG15. ** *p* < 0.01 *** *p* < 0.001. Fold changes are based on GAPDH as control.

We tested whether MYXV infection prior to the treatment of cells with type I IFN might block subsequent type I IFN signaling in virus-infected rabbit, human or mouse cells. For this, cells were first infected with either WT-MYXV or vMyxM029KO viruses with an MOI of 5 for 6 h and then followed by addition of type I IFN. In these pre-infected cells, the effects of subsequent type I IFN treatment on virus replication was measured by tittering progeny. Our results indicate that both the WT-MYXV and vMyxM029KO viruses were able to complete their replication cycles in RK13, HeLa PKR-/- or MEF PKR-/-cells even in the continued presence of type I IFNs starting at 6 h (Figure 9A). This was further confirmed by calculating the log_10_ changes in virus titer, which showed very minimal changes in virus titer (Figure 9B).

These results suggest that M029 is not required for the viral inhibition of type I IFN signaling that establishes the antiviral state. To further confirm this, we checked the level of STAT-1 phosphorylation after treatment with hIFNβ in the absence or presence of MYXV infection of HeLa cells. Phosphorylated STAT1 (pSTAT1) can be detected only in the IFNβ-treated cells but not in WT-MYXV or vMyx-M029KO infected cells (Figure 9C). Infection with either WT-MYXV or vMyxM029KO viruses before hIFNβ treatment equivalently reduced the levels of pSTAT1 after 6 h of infection, but not at 1 h. This suggests that M029 is not the viral protein involved in the inhibition of IFN ligand-induced signaling after MYXV infection.

To further confirm that WT-MYXV or vMyxM029KO infection prior to the treatment with type I IFN can block subsequent IFN-induced STAT signaling, we examined the levels of IFN-induced ISGs in different cell types. Both WT-MYXV and M029-minus virus significantly reduced the level of IFN-induced expression of ISGs like Mx1 and ISG15 in human and mouse cell lines, as measured by qPCR (Figure 9 D,E). These results confirm that MYXV infection, even in the absence of M029, inhibited IFN-induced STAT signaling and thus also the induction of downstream ISGs, provided that the cells are pre-infected prior to IFN addition.

## 4. Discussion

In this study we describe the role of the MYXV-encoded dsRNA binding protein M029 in antagonizing the antiviral state induced by type I IFNs in different species of host cells. This is of particular interest because, although the natural in vivo tropism of MYXV is strictly for lagomorphs such as the European rabbit, the virus is also permissive in cultured cells derived from many diverse non-rabbit species in vitro. Indeed, MYXV replicates productively in many classes of human cancer cells and is being developed as an oncolytic viro-therapeutic for several diverse human cancers [10,12]. M029 is a pivotal host range protein that is required for MYXV replication in a broad variety of cultured cell lines originated from diverse species including rabbits, human and mice. We have previously reported that at least some of the host range functions of M029 are mediated by the inhibition of PKR activation/phosphorylation in response to virus infection [26]. In all human cells that were tested, siRNA-mediated knockdown of the expression of PKR at least partially rescued the replication defect of M029-minus MYXV. We now show that for both human and mouse cells, the replication of M029-minus virus can be rescued in part by genetic knockout of PKR in these cells. These observations suggest that the host range tropism function of M029 is mediated in part by the inhibition of PKR activation in a relatively species-independent manner. We also report that, although PKR inhibition is critical for MYXV tropism in transformed/immortalized human or mouse cells, important additional antiviral functions of M029 are mediated independently of PKR and this latter activity of M029 is remarkably species specific. Specifically, we show that MYXV exploits M029 to achieve close to total nullification of the type I IFN induced antiviral state in rabbit cells, but this IFN blockade is only partial against the antiviral state established in human cells and is nearly nonexistent against the antiviral state in mouse cells.

Type I IFNs play a major role in inducing innate antiviral responses that protect primary cells and tissues from virus infection. To establish successful infection in their hosts, many viruses are able to antagonize one or more aspects of the type I IFN-induced antiviral responses. MYXV is a rabbit specific poxvirus and inhibits rabbit type I IFN induced responses as part of its genetic program that mediates pathogenesis in the European rabbit host. However, at the molecular level, how MYXV neutralizes the type I IFN pathway and subsequent IFN-induced antiviral state remains to be clarified. MYXV lacks an obvious encoded type I IFN receptor homolog, such as the secreted B18 IFN antagonist in VACV [34]. Although the MYXV ORF 135 is a related family member to other poxviral homologs of the IFN receptor, subsequent studies failed to demonstrate any anti-IFN activities of M135 in rabbits or in rabbit cells [31]. Later studies using rabbit RK13 cells demonstrated that MYXV is indeed able to effectively inhibit rabbit IFN responses, which suggested that MYXV likely encodes protein(s) that are able to inhibit IFN signaling and/or the IFN-induced antiviral state. We now demonstrate that the ability of MYXV to inhibit type I IFN responses in rabbit cells is either compromised or completely lost when the virus infects human or mouse cells possessing IFN-response competency.

In rabbit RK13 cells, M029-minus MYXV can still replicate and produce infectious progeny following high multiplicity infection, but the foci size after low multiplicity infection was smaller compare to the WT-MYXV infection [26]. This is partly because MYXV-encoded protein M156, a homologue of VACV K3, can inhibit rabbit PKR in RK13 cells [35]. However, we now show that type I IFN pretreatment of RK13 cells and induction of the antiviral state completely inhibited late MYXV protein synthesis in the absence of M029, which then prevented the formation of progeny. However, the M029-minus MYXV was still able to inhibit the IFN signaling induced by exogenous IFN ligand added 6 hpi even in the absence of M029. This suggests that other protein(s) independent of M029 from MYXV are involved in the blockade of IFN-induced JAK/STAT signaling. Interestingly, unlike WT-MYXV, M029-minus virus could not spread efficiently to neighboring uninfected RK13 cells and form foci, thus further confirming that M029 is required for antagonizing the induced antiviral state in rabbit cells.

MYXV is able to successfully replicate in some cultured human cells, such as human primary fibroblasts, as long as the infected cells do not induce IFN and/or TNF as a consequence of the virus infection. Thus, MYXV is non-permissive in primary human macrophages because the virus infection co-induces both IFN and TNF, that rapidly renders cells in the culture (including even admixed “permissive” human fibroblasts) non-permissive for the virus in a paracrine fashion. However, unlike rabbit cells, where the viral inhibition of IFN pathways is very successful, MYXV is able to only partially antagonize the human type I IFN induced antiviral state [6,36]. Here we show that the ability of MYXV to replicate in human cells is compromised in the presence of type I IFN, provided the cells can respond to IFN, but this partial inhibition is totally lost in the absence of M029. This suggests that M029 has retained the ability to partially antagonize the IFN-induced antiviral state in human cells, albeit with much less efficiency than in rabbit cells. Interestingly, even in the presence of M029, MYXV is not able to spread and infect neighboring human cells when the cells are pretreated with IFN. Furthermore, in human cells, viral gene expression analysis suggests that although IFN-induced activities severely compromise viral gene expression, there was still detectable expression of some late viral proteins, which subsequently allowed lower but still detectable levels of progeny virus formation.

However, as found in rabbit cells, MYXV is also able to efficiently inhibit IFN signaling in human cells as long as the virus infection is established prior to the addition of IFNs. Our current and previous results revealed that MYXV infection caused dephosphorylation of Type I IFN signaling proteins STAT1 and Tyk2 in human cells [36]. These observations suggested that a viral phosphatase may be involved in the dephosphorylation of these IFN signaling molecules. In VACV, the viral phosphatase VH1 is delivered by the input virions, which causes a rapid dose dependent inhibition of either the type I or type II IFN signal transduction [37,38]. Since we did not observe any substantial decrease in the phosphorylation of STAT1 after 1 h infection with either WT-MYXV or M029-minus viruses, but inhibition of STAT1 activation is robust by 6 hpi, this suggests that for MYXV the viral phosphatase may not be encapsidated within the virion. Since we observed significant decrease in phosphorylation of STAT1 and expression of downstream ISGs after 6 h of infection with either WT-MYXV or the M029-knockout virus, this suggests that inhibition of IFN signaling in MYXV-infected cells also blocked the expression of ISGs and their possible antiviral effects in these infected cells.

Outside the rabbit, MYXV modulation of the type I IFN pathway is highly dependent on the infected cell type. In primary MEFs, MYXV infection is rapidly sensed and induces the activation of IFN signaling pathway via phosphorylation of the mitogen-activated protein kinase ERK1/2, which induces type I IFN and thus inhibits the further replication of MYXV [7]. However, when the MEFs are immortalized, even after as few as 10 passages in culture (iMEFs), MYXV is now able to productively infect the cells and produce progeny [8]. This suggests that as the MEFs are immortalized they lose their innate ability to sense MYXV and no longer induce IFN in response to the infection, although the cells still possess functionally intact IFN signaling pathways (and still become non-permissive to MYXV following addition of exogeneous IFN). Thus, the mouse type I IFN-induced antiviral state still totally inhibits WT-MYXV replication in MEFs, unlike rabbit cells. The replication of M029-minus virus in iMEFs can be rescued only after PKR knockdown or knockout, however this absence of PKR is still not sufficient to antagonize the IFN-induced antiviral state against either WT-MYXV or vMyxM029KO viruses. Our results clearly indicate that the MYXV proteins involved in circumventing the antiviral state are operationally nonfunctional in mouse cells and only partially functional in human cells. Interestingly, even in MEFs or MEF PKR-/-, MYXV or M029-minus virus infection can still efficiently inhibit mouse type I IFN-induced signaling provided the IFN is added at least 6 h after the viral infection, suggesting that the induced viral protein(s) are involved in blocking IFN signaling downstream of the IFN receptor. The most likely candidate is the viral dual specificity phosphatase, encoded by M069, which operates in a species independent manner.

This is a first study showing that a viral dsRNA binding protein can mediate antiviral functions in cells from multiple species, but exhibits only strict species-specific inhibition of the IFN-induced antiviral state (in this case, fully in rabbit cells, partially in human cells, and not at all in mouse cells). In contrast, MYXV is able to inhibit IFN signaling in a species-independent manner following virus infection of cells, even in the absence of a soluble decoy receptor for type I IFN, but is highly species-specific in its ability to antagonize the pre-existing antiviral state.

The prototypic poxvirus VACV antagonize type I IFN activities at both the extracellular and intracellular levels. VACV type I IFN secreted binding protein B18 competes for IFN binding and activating of host cellular IFNRs [34]. Other VACV intracellular proteins that modulate IFN signaling or specific IFN activities include VH1, E3, K3, K1, C6 and C7 [19,37,38,39,40]. To date, no known functional counterpart of the secreted B18 IFN antagonist has been identified in MYXV that inhibits extracellular rabbit IFN. But relatives of some of the other intracellular VACV anti-IFN effectors, like VH1, E3, K3 and C7, are indeed encoded by MYXV. This paper presents the first report of a MYXV-encoded intracellular modulator of IFN activities, M029, which functions to coordinately modulate several host response pathways, some of which are highly rabbit-specific, whereas others are independent of the host species. Reports indicate that VACV E3 inhibits IFN-induced responses by inhibiting PKR and also by directly targeting ISG15, which is induced by type I IFN. Our results indicate that M029 also inhibits IFN activities by targeting functions of at least some ISGs, however, unlike E3, M029 inhibits the IFN-induced antiviral state independently of PKR. In our observations, even the WT-MYXV is unable to alter the pre-induced expression levels of ISGs in all the cell types that we have tested, provided the antiviral state is established prior to virus infection. Furthermore, transient expression of M029 alone did not block either the transcription or translation of any tested ISGs. Instead, M029 targets not only ISG effector functions responsible for the antiviral state but also intrinsic antiviral factors that are constitutively present and otherwise inhibit MYXV replication in non-rabbit cells in the absence of M029 (data not shown). Instead, MYXV gene products, of which M029 is a major player, can completely overcome the IFN-induced antiviral state in rabbit cells, but only partially in human cells, and not at all in mouse cells. In human cancer cells that have undergone multiple defects in their capacity to mount antiviral defense pathways, there is an even further transition away from the primary human cell sensitivity to IFN to a more “rabbit-like” phenotype that is more susceptible to infection by MYXV.

IFN induces the expression of hundreds of ISGs in all mammalian cells, but it is not known how many of these actually possess operational antiviral functions against poxviruses like MYXV, nor how these antiviral activities compare between species. At this point it is difficult to define how many host ISGs are actually targeted by M029. It is clear that host PKR is targeted for inhibition by M029 to mediate tropism in mammalian cells from many species but M029 also plays a key role in the inhibition of the antiviral state induced by IFN in rabbit cells, a partial inhibitory role in human cells, and essentially no inhibitory role in mouse cells. The M029KO virus remains sensitive to type I IFN-induced responses even in the absence of PKR, suggesting that other cellular ISG proteins are also targeted for inhibition of the IFN-induced antiviral state, which is probably linked with the host specific modulatory functions of these members of the E3 family of RNA binding proteins. Thus, replacing any one viral dsRNA binding protein with even a related orthologue from a different virus may rescue the in vitro replication functions in certain cultured cells but not necessarily restore full in vivo pathogenesis.

Our results also indicate that MYXV is able to inhibit type I IFN signaling in most cell types, once the cell is infected and has had sufficient time to express its repertoire of early immunoregulatory proteins. However, MYXV is severely defective at neutralizing a pre-existing IFN-induced antiviral state in cells outside of the rabbit species, which indicates that many of the viral immune-inhibitory molecules are more adapted for the rabbit species-specific inhibition of type I IFN. Like VACV, MYXV almost assuredly encodes additional proteins that function in the inhibition of the IFN antiviral state, in addition to M029. Further knowledge about how MYXV manipulates IFN pathways in human cells, in particular for primary vs transformed cells, will help further the development of MYXV as oncolytic virus for the treatment of human cancers. These studies will also enhance our knowledge about viral tropism at the cellular and host level, not only in evolutionary hosts, but also when a virus occasionally makes a species leap into a new host.
